# Primary malignant melanoma of the lung with* C-KIT* mutation and *SRD5A3-KIT* fusion

**DOI:** 10.1186/s13000-025-01711-5

**Published:** 2025-10-14

**Authors:** Lan Shen, Pei Guo, Mingzhen Li, Ting Jiang, Anjia Han, Xiaojuan Pei

**Affiliations:** 1 Department of Pathology, Shenzhen Hospital of Southern Medical University, 13 Xinhu Road, Bao’an District, Shenzhen City, 518110 China; 2 Department of Pathology, Shanghai Jiao Tong University Affiliated Sixth Peoples Hospital, Shanghai, China; 3 Department of Pathology, the First Affiliated Hospital, Sun Yat-Sen University, Guangzhou, China

**Keywords:** Lung Primary melanoma, c-KIT mutation, SRD5A3-KIT fusion

## Abstract

**Background:**

Primary pulmonary malignant melanoma (PMML), an exceedingly rare aggressive neoplasm originating from bronchial mucosal melanocytes, is characterized by early metastatic dissemination and high mortality. While over 95% of malignant melanomas are cutaneous in origin, fewer than 80 PMML cases have been documented globally. The molecular pathogenesis of PMML remains poorly defined, with less prior genomic studies utilizing Next-generation sequencing (NGS) reported to date.

**Case Presentation:**

A 68-year-old asymptomatic woman was referred to our institution in June 2022 after a routine health screening revealed a solitary pulmonary nodule. Chest CT demonstrated a 1.2 cm × 0.8 cm hypodense nodular opacity nodule in the posterior segment of the left upper lobe. The lesion remained stable during a 2-month observation period. Despite the absence of respiratory symptoms (e.g., cough, hemoptysis) or constitutional signs (e.g., weight loss), the patient elected surgical resection due to persistent malignancy concerns.

**Conclusion:**

Histopathological examination revealed tumor cells exhibiting epithelioid to spindle-shaped morphology, characterized by prominent nucleoli and intracytoplasmic melanin deposition (hematoxylin and eosin staining). Immunohistochemical analysis demonstrated diffuse and strong positivity for S-100, HMB-45, and Melan-A. Based on the histomorphological features and immunohistochemical profile, a diagnosis of malignant melanoma was established. NGS detected a somatic *KIT* exon 11 mutation (c.1727 T > C, p. Leu576Pro; variant allele frequency: 20.1%) and identified an *SRD5A3-KIT* gene fusion involving transcript variants NM_024592.4 (*SRD5A3)* and NM_000222.2 (*KIT*), with breakpoints in Exon 5 of *SRD5A3* and Exon 6 of *KIT*. The fusion variant showed a somatic mutation frequency of 24.8%. These findings not only expand the molecular landscape of PMML but also suggest therapeutic opportunities through targeted kinase inhibition. This case underscores the critical role of integrated multimodal analysis (radiological-pathological-molecular) in characterizing rare malignancies.

**Supplementary Information:**

The online version contains supplementary material available at 10.1186/s13000-025-01711-5.

## Background

Malignant melanoma is a highly aggressive non-epithelial tumor. Studies have shown that its incidence does not differ by gender and that it primarily arises in the skin, particularly in the lower limbs. However, it can also develop in mucosal tissues, such as those of the oral cavity, anorectum, vagina, and esophagus [1]. PMML represents an exceptionally rare pulmonary malignancy, comprising approximately 0.01% of all primary lung tumors and 0.4% of malignant melanomas. To date, only 76 cases have been documented in the literature, with molecular characterization available in 11 cases [2, 3]. Notably, only one was found to harbor a *BRAF* mutation, and one was identified with an *NRAS* mutation [3, 4]. We report a case of a 68-year-old woman who fulfilled the relevant diagnostic criteria for PMLL. This report characterizes the imaging, histopathological, and molecular alteration s observed in this case.

## Case presentation

**Fig. 1:**
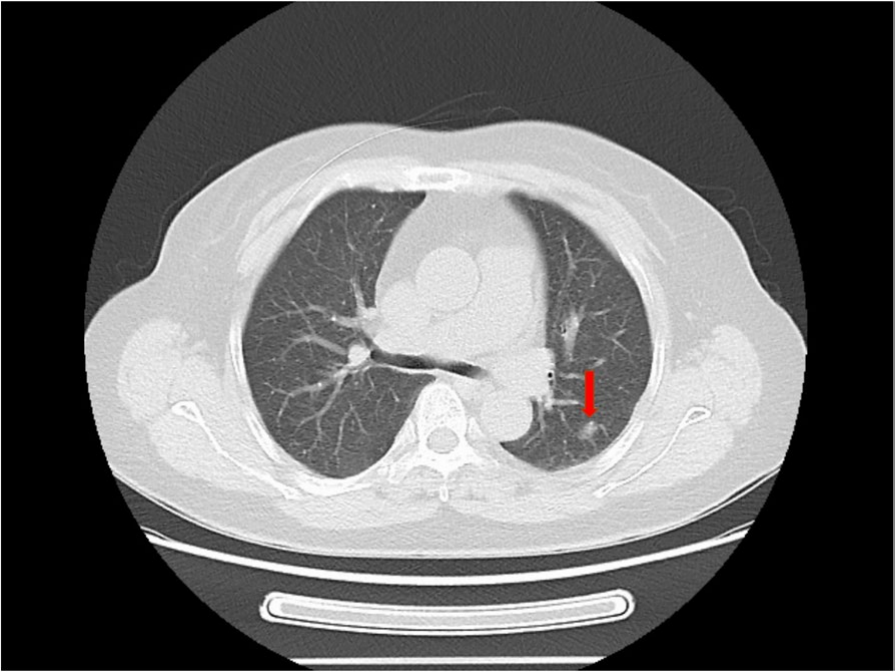
Chest computed tomography (CT) revealed a (1.2 × 0.8 cm), heterogeneous, hypodense nodule in the apico-posterior segment of the left upper lobe (red arrow, Fig. 1), demonstrating heterogeneous attenuation without calcification or cavitation

**Fig. 2:**
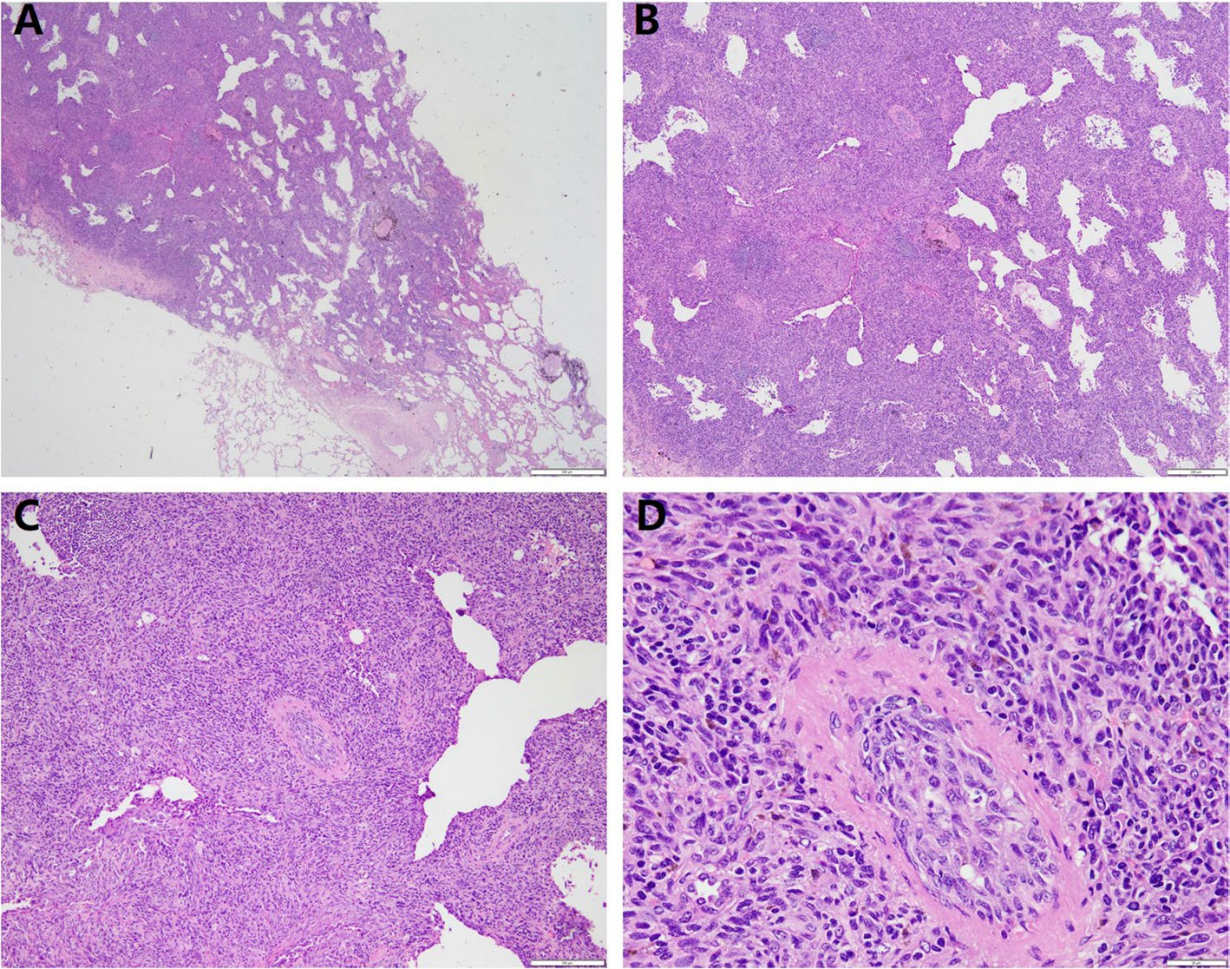
The tumour is poorly demarcated from the surrounding area and grows along the alveolar wall (**A**). The tumour is mostly solid with some glandular lumen-like structures (**B**). The tumour in the solid area consists of epithelioid cells growing in nests and spindle cells arranged in bundles, while tumour cells are seen to invade the blood vessels (**C**). The tumour cells are clearly heterogeneous with distinct nucleoli, pathological nuclear divisions are easily seen, deposits of melanin granules are seen and eosinophilic infiltration is seen in the interstitium (**D**)

**Fig. 3:**
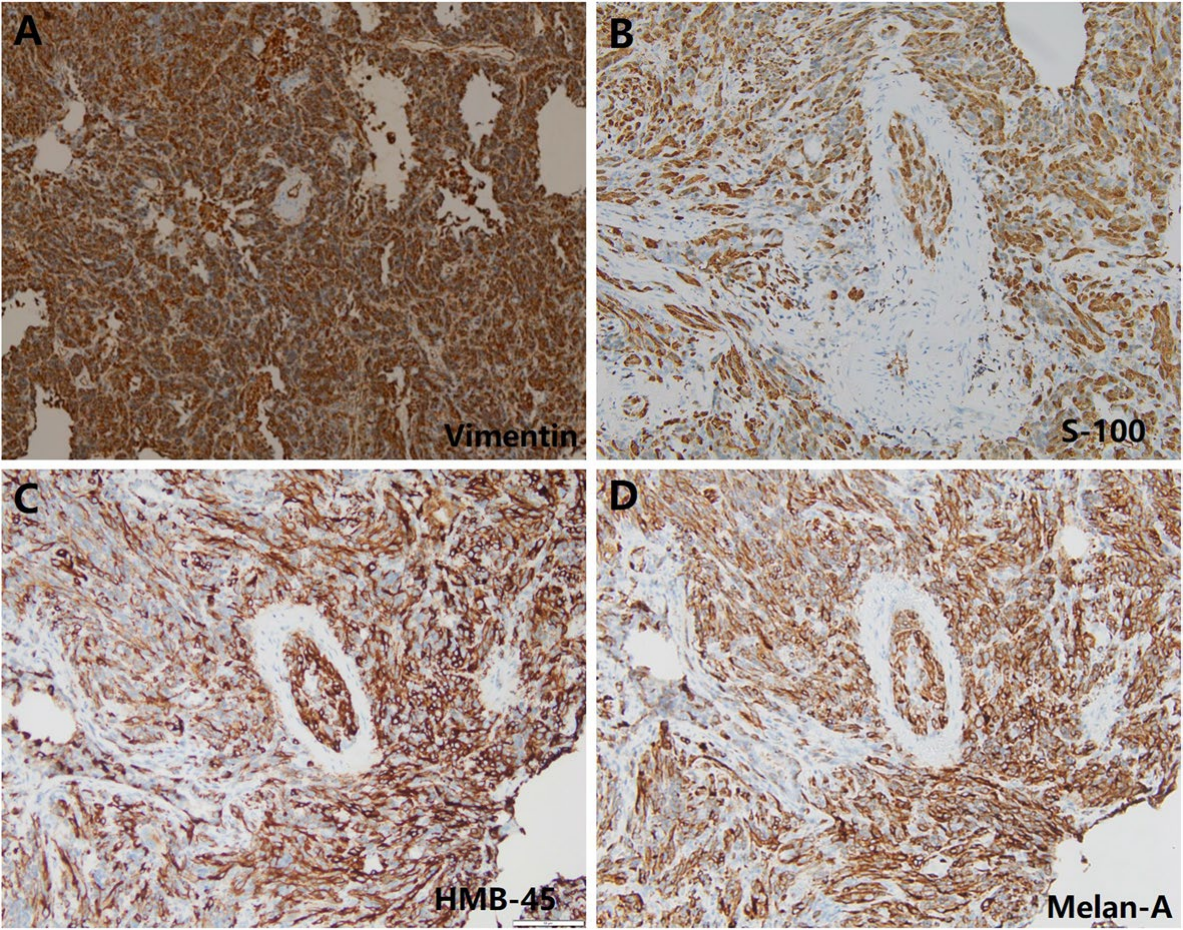
Immunohistochemical features of lung tissue biopsy Positive cytoplasmic staining of the tumor cells for Vimentin (**A**), S100 (**B**), MelanA (**C**), and HMB-45 (**D**)

**Fig. 4:**
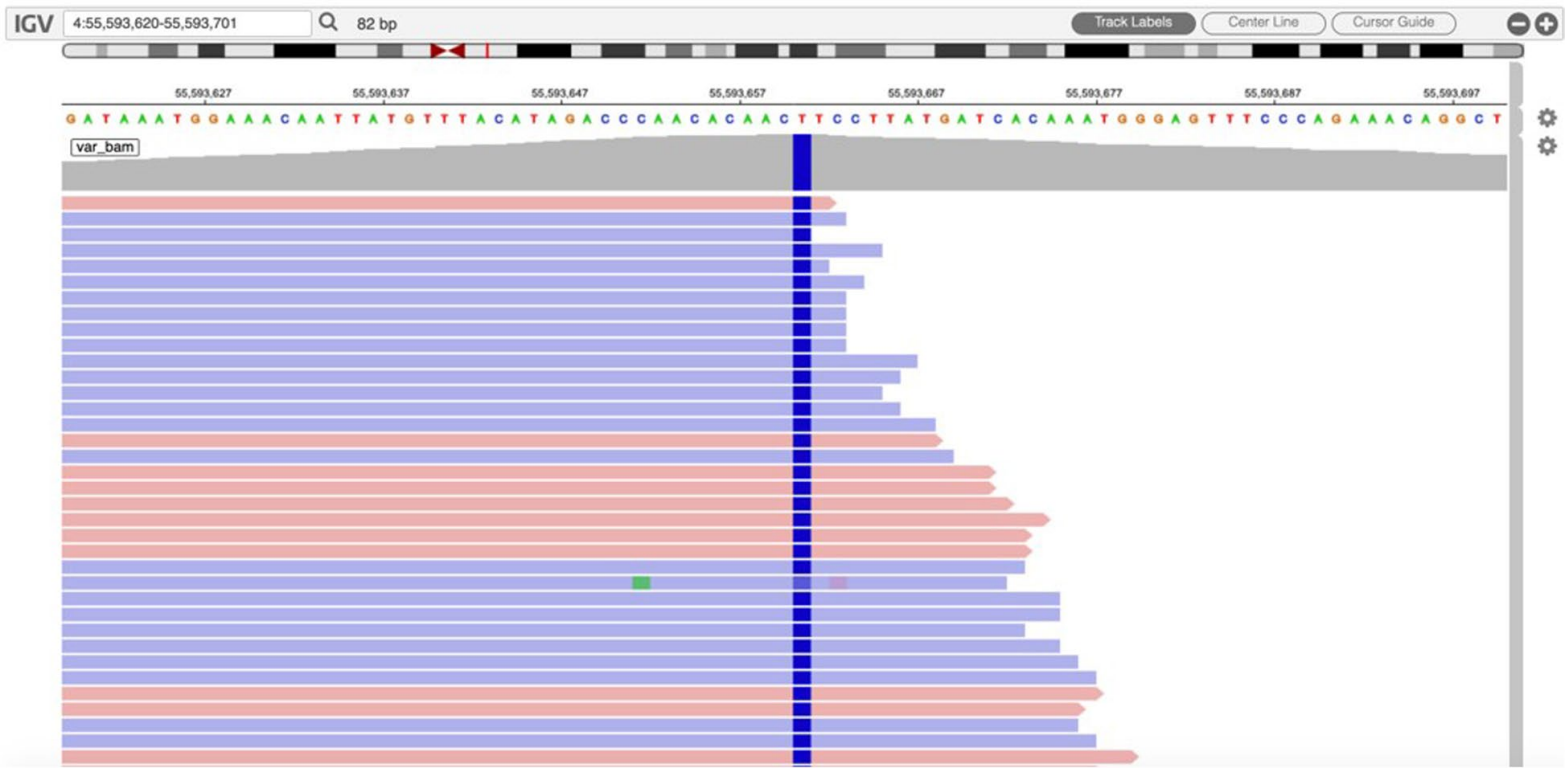
NGS detected a somatic KIT exon 11 mutation (c.1727 T > C, p. Leu576Pro; variant allele frequency: 20.1%)

We report a case of a 68-year-old woman who presented with a pulmonary nodule identified by imaging at our hospital in June 2022.The patient was asymptomatic, with no respiratory complaints such as cough, sputum production, or hemoptysis. Chest computed tomography (CT) revealed a (1.2 × 0.8 cm), heterogeneous, hypodense nodule in the apico-posterior segment of the left upper lobe (Fig. 1). To establish a definitive diagnosis, a wedge resection of the affected lung tissue was performed. Histopathological examination of hematoxylin–eosin (H&E)-stained sections demonstrated tumor cells with prominent intracytoplasmic and interstitial melanin deposition (Fig. 2). Immunohistochemical analysis further supported the diagnosis, revealing strong positivity for melanoma markers (HMB-45, Melan-A, and S-100) and vimentin (Fig. 3). In addition, primary lesions in areas such as the skin, anorectum, vagina, and esophagus have been excluded through systematic screening, thus confirming the diagnosis of primary pulmonary malignant melanoma (PMML).confirming primary pulmonary malignant melanoma (PMML). Molecular characterization by NGS detected a somatic *KIT* exon 11 mutation (c.1727 T > C, p. Leu576Pro; variant allele frequency: 20.1%) and identified an *SRD5A3-KIT* gene fusion involving transcript variants NM_024592.4 (*SRD5A3*) and NM_000222.2 (*KIT*), with breakpoints in Exon 5 of *SRD5A3* and Exon 6 of KIT  (Fig. 4). The fusion variant showed a somatic mutation frequency of 24.8%. This specific rearrangement has not been previously reported in the literature, and its functional implications remain to be elucidated. Additional genomic alterations included amplifications of *KDR, KRAS, ERBB3, CDK4,* and *MDM2*. Notably, no pathogenic variants were detected in commonly mutated melanoma-associated genes (*BRAF, NRAS,* or *TP53*).

## Discussion and conclusions

Malignant melanoma, which predominantly originates in the skin, may develop at any anatomical site and across all age groups. Existing literature indicates that primary malignant melanoma of the lung (PMLL) predominantly affects middle-aged and elderly populations, demonstrating a median age of approximately 60 years with a notable male predilection [3]. In our case, there were no associated clinical signs, such as cough, hemoptysis, pneumonia, or atelectasis, and the lung lesion was found incidentally during a routine examination.

The principal diagnostic challenge lies in differentiating PMML from metastatic pulmonary melanoma. The widely accepted diagnostic criteria established by Jensen and Egedorf require fulfillment of four essential components: i) radiographic evidence of an isolated pulmonary mass or nodule; ii) histopathological confirmation through characteristic morphological features accompanied by intracellular melanin granules verified via immunohistochemical markers (S-100, HMB-45, Melan-A) or ultrastructural analysis; iii) absence of prior melanoma excisions from cutaneous or mucosal surfaces, except when histopathological review conclusively excludes primary melanoma; and iv) lack of detectable extrathoracic melanoma upon initial diagnosis [5]. In the current case, radiographic detection of an asymptomatic pulmonary lesion preceded histopathological confirmation of malignant melanoma exhibiting typical morphological characteristics. Immunohistochemical profiling demonstrated strong positivity for S-100, HMB-45, and Melan-A markers. Comprehensive postoperative evaluation revealed no primary lesions in cutaneous, anorectal, vaginal, or esophageal regions, thereby satisfying all established diagnostic criteria for PMML.

Malignant melanoma demonstrates high tumor aggressiveness and carries a significant tumor mutational burden. The four predominant mutational subtypes comprise *BRAF* mutations (35%-50%), *RAS* mutations (10%-25%), *NF1* mutations (14%), and triple wild-type (WT) tumors, as documented in current genomic classification [6, 7]. Specifically, the B*RAF V600E* variant accounts for 87.3% of *BRAF*-driven cases, while *RAS* mutations primarily localize to codon 61 (exon 3) and codon 12 (exon 2), exhibiting an overall prevalence of 12.6%. In contrast, *C-KIT* mutations display lower frequency (9.4% in cohort studies), with the L576 residue in exon 11 representing the most recurrent alteration [8]. Regarding primary pulmonary malignant melanoma, existing molecular evidence remains limited. Sporadic reports describe *TP53* mutations in pulmonary melanoma [9], while *NRAS* mutations have been occasionally observed [4]. In the current case, high-throughput sequencing revealed a pathogenic *C-KIT* L576P mutation (exon 11) accompanied by a *SRD5A3-KIT* fusion gene. To our knowledge, this specific genomic configuration has not been previously reported, and its functional implications remain undetermined. Additional genomic amplifications included *KDR, KRAS, ERBB3, CDK4,* and *MDM2*. Notably, no mutations were detected in *BRAF, NRAS,* or *TP53* hotspots.

Current treatment paradigms for primary malignant melanoma of the lung (PMML) lack standardized protocols, with complete surgical resection combined with regional lymphadenectomy constituting the mainstay of management [3]. In advanced cases with distant metastases, adjuvant systemic therapies frequently yield suboptimal clinical outcomes, as evidenced by limited therapeutic responses in reported series [10, 11]. Emerging molecular therapeutics present new opportunities, given melanoma's well-documented susceptibility to targeted therapies. Of particular significance, BRAF inhibitors (Vemurafenib) demonstrate substantial efficacy in *BRAF*-mutated cutaneous melanomas (objective response rates: 48–53%) [12], while tyrosine kinase inhibitors (e.g., imatinib) achieve clinical benefit in *c-KIT*-driven subtypes. Notably, the applicability of these regimens to PMML remains unexplored in controlled trials, with only anecdotal evidence available from case reports [13]. In the present case, definitive surgical intervention (wedge resection) was performed without adjuvant pharmacological therapy. During the 18-month follow-up period, serial imaging surveillance revealed no evidence of local recurrence or metastatic progression. This favorable prognosis contrasts with the generally poor outcomes documented in historical PMML cases, suggesting potential prognostic heterogeneity within this rare disease entity.

In summary, Primary malignant melanoma of the lung (PMML) represents a rare clinical entity that requires rigorous differentiation from metastatic melanoma. Definitive diagnosis relies on histopathological evaluation supplemented by immunohistochemical profiling (S-100, HMB-45, Melan-A), which remain the diagnostic gold standard. Emerging molecular diagnostics have revolutionized oncology practice, providing critical insights into tumor molecular subtyping, personalized therapeutic strategies, and prognostic stratification. While significant advancements have been achieved in targeted therapy for cutaneous melanoma (e.g., BRAF inhibitors, anti-KIT agents), the absence of standardized diagnostic and therapeutic guidelines for PMML persists. Current clinical management predominantly extrapolates from cutaneous melanoma protocols, with therapeutic agent selection guided by tumor molecular profiling.

Notably, the scarcity of reported PMML cases with comprehensive molecular characterization significantly limits evidence-based decision-making. This case contributes novel molecular insights, particularly the identification of a previously unreported *SRD5A3-KIT* fusion alongside amplifications in *KDR, KRAS*, and other oncogenic drivers. Such findings underscore the necessity for systematic molecular profiling in PMML to elucidate pathogenesis and optimize therapeutic interventions. Further multicentric studies are imperative to establish diagnostic criteria and evaluate targeted therapy efficacy in this enigmatic malignancy.

## Supplementary Information

Supplementary Material 1.

## Data Availability

No datasets were generated or analysed during the current study.

## Abbreviations

PMML: Primary malignant melanoma of the lung
NGS: Next-generation sequencing
CT: Computed tomography

## References

[1] Kapatia G, Gupta P, Rohilla M, et al. The spectrum of malignant melanoma on cytology: a tertiary care center study. Diagn Cytopathol. 2019;47:1018–23. 10.1002/dc.24265. Journal Article. 31260174 10.1002/dc.24265

[2] Wilson RW, Moran CA. Primary melanoma of the lung: a clinicopathologic and immunohistochemical study of eight cases. Am J Surg Pathol. 1997;21:1196–202. 10.1097/00000478-199710000-00010. Journal Article. 9331292 10.1097/00000478-199710000-00010

[3] Paliogiannis P, Fara AM, Pintus G, et al. Primary melanoma of the lung: a systematic review. Medicina (Kaunas). 2020. 10.3390/medicina56110576. 33142971 10.3390/medicina56110576 PMC7693850

[4] Hibiya T, Tanaka M, Matsumura M, et al. An NRAS mutation in primary malignant melanoma of the lung: a case report. Diagn Pathol. 2020;15:11. 10.1186/s13000-020-0928-8. Case Reports; Journal Article. 32028967 10.1186/s13000-020-0928-8 PMC7006422

[5] Jensen OA, Egedorf J. Primary malignant melanoma of the lung. Scand J Respir Dis. 1967;48:127–35. Journal Article. 5183297

[6] Genomic Classification of Cutaneous Melanoma. *Cell* 2015; 161: 1681–1696. Journal Article; Research Support, N.I.H., Extramural. 10.1016/j.cell.2015.05.044. 10.1016/j.cell.2015.05.044 PMC4580370 26091043

[7] Leichsenring J, Stögbauer F, Volckmar AL, et al. Genetic profiling of melanoma in routine diagnostics: assay performance and molecular characteristics in a consecutive series of 274 cases. Pathology. 2018;50:703–10. 10.1016/j.pathol.2018.08.004. Journal Article. 30348504 10.1016/j.pathol.2018.08.004

[8] Ren M, Zhang J, Kong Y, et al. BRAF, C-KIT, and NRAS mutations correlated with different clinicopathological features: an analysis of 691 melanoma patients from a single center. Ann Transl Med. 2022;10:31. 10.21037/atm-21-4235. 35282092 10.21037/atm-21-4235 PMC8848432

[9] Watanabe M, Yamamoto H, Hashida S, et al. Primary pulmonary melanoma: a report of two cases. World J Surg Oncol. 2015;13:274. 10.1186/s12957-015-0695-2. Case Reports; Journal Article. 26376781 10.1186/s12957-015-0695-2 PMC4573480

[10] Shi Y, Bing Z, Xu X, et al. Primary pulmonary malignant melanoma: Case report and literature review. Thorac Cancer. 2018;9:1185–9. 10.1111/1759-7714.12798. Case Reports; Journal Article; Review. 30062692 10.1111/1759-7714.12798 PMC6119615

[11] Nigi A, Toyoshima H, Kondo S, et al. Primary melanoma in the bronchus. Clin Case Rep. 2021;9:e5192. 10.1002/ccr3.5192. Journal Article. 10.1002/ccr3.5192 PMC8659558 34938558

[12] Imianitov EN. Melanoma: from molecular studies to the treatment breakthrough. Arkh Patol. 2013;75:63–72. Journal Article; Review. 24341237

[13] Held L, Eigentler TK, Meier F, et al. Oncogenetics of melanoma: basisfor molecular diagnostics and therapy. J Dtsch Dermatol Ges. 2011;9:510–6. 10.1111/j.1610-0387.2011.07603.x. Journal Article; Review. 21244632 10.1111/j.1610-0387.2011.07603.x

