# Supplementary material for: Primary malignant melanoma of the lung with C-KIT mutation and SRD5A3-KIT fusion
Source: Diagn Pathol. 2025 Oct 14;20:113. doi: 10.1186/s13000-025-01711-5 (PMC12522767; doi:10.1186/s13000-025-01711-5)
Supplement: Supplementary file 1 — Supplementary Material 1. [file 13000_2025_1711_MOESM1_ESM.pdf]

Next-Generation Sequencing (NGS) Methodology

1. Reagents and Instruments

1.1 DNA Extraction Kits:

(1) Tumor tissue genomic DNA: QIAamp DNA Mini Kit (Qiagen, Germany)

(2) Peripheral blood lymphocytes (PBLs): DNeasy Blood & Tissue Kit (Qiagen, Germany)

(3) Plasma circulating nucleic acids (cfDNA): QIAamp Circulating Nucleic Acid Kit (Qiagen, Hilden, Germany)

1.2 Quality Control Equipment

(1) DNA concentration measurement: Qubit dsDNA HS Assay Kit (ThermoFisher)

(2) DNA integrity analysis: Agilent 2100 BioAnalyzer (Agilent Technologies, Santa Clara, CA, USA)

1.3 Library Preparation

(1) Fragmentation: Covaris S2 (Woburn, MA, USA)

(2) Library construction: KAPA Library Preparation Kit (KAPA Biosystems, Wilmington, MA, USA)

(3) Hybridization capture probes: Custom biotinylated oligonucleotide probes (IDT, Coralville, IA, USA)

1.4 Sequencing Platform

(1) MGIseq-2000 sequencer (BGI, Shenzhen, China)

(2) Sequencing technology: Combinatorial Probe-Anchor Synthesis (cPAS)

2.Sample Processing and Quality Control

2.1 Sample Types

(1) Baseline tumor tissue (FFPE)

(2) Peripheral blood (pre-treatment and post-neoadjuvant therapy time points): 20 mL whole blood collected (Streck cfDNA blood collection tubes)

2.2 Nucleic Acid Extraction

(1) Plasma separation: Double centrifugation (2500g × 10 min → 16000g × 10 min)

(2) DNA quality control standards:Concentration ≥2.5 ng/μL (Qubit measurement);Fragment distribution: 200-250 bp (Agilent 2100 HS DNA Kit)

3. Library Preparation and Hybridization Capture

3.1 Library Construction

(1) DNA fragmentation: Covaris S2 shearing to 200-250 bp

(2) End repair/adapter ligation: KAPA kit with unique identifiers (UIDs)

3.2 Hybridization capture:

(1) Targeting 1021 cancer-related genes (SeqCap EZ Library System, Roche NimbleGen)

(2) Liquid-phase hybridization with biotinylated probes (65°C, 16 hours)

(3) Streptavidin bead enrichment of target regions

3.3 Sequencing Parameters

(1) Platform: MGIseq-2000

(2) Read length: 150 bp paired-end sequencing

(3) Data generation: DNA nanoballs (DNBs) loaded onto patterned array chips, imaged using cPAS technology

4. Data Analysis Pipeline

4.1 Raw Data Processing

(1)Low-quality read filtering (NCfilter software):Adapter contamination, >50% low-quality bases ($Q \leqslant 5$), N rate >10%;Reference genome alignment: BWA v0.6.2 (hg19/GRCh37);Duplicate marking: Picard MarkDuplicates (PBLs); UID error correction (realSeq)

(2) Quality recalibration: GATK IndelRealignment + BaseRecalibrator

4.2 Variant Detection

(1) Somatic SNVs/Indels: MuTect algorithm;Filtering criteria:Germline mutations (PBLs control)

(2) Database filtering (dbSNP, 1000 Genomes, ExAC):Supporting reads: ⩾5 reads for tissue driver genes (mapQ/baseQ⩾30);ctDNA Positivity Criteria;Tumor-informed analysis: Matching tissue variants

4.3Supporting read thresholds:

(1)Tissue driver genes: ⩾2 reads; non-recurrent variants: ⩾4 reads

(2)Hotspot variants: ⩾4 reads; non-hotspots: ⩾8 read

5.Data Availability

5.1 Raw data: NCBI SRA (SRR23910631)

5.2Analysis pipeline: BioProject PRJNA946381
